# Cocreating a programme to prevent injuries and improve performance in Australian Police Force recruits: a study protocol

**DOI:** 10.1136/bmjsem-2025-002531

**Published:** 2025-03-12

**Authors:** Myles C Murphy, Andrea B Mosler, Jonathan Hodgson, Sophia Nimphius, Evert Verhagen, Joanne Kemp, Alex Donaldson, Debra Langridge, Vanessa R Sutton, Kay M Crossley, Clare L Ardern, Carolyn A Emery, Mary A Kennedy, Simone Radavelli-Bagatini, Martin Hägglund, Brady Green, G Gregory Haff, Garth Allen, Andrea Bruder

**Affiliations:** 1 Nutrition and Health Innovation Research Institute, School of Medical and Health Sciences, Edith Cowan University, Joondalup, Perth, Australia; 2 School of Health Sciences, The University of Notre Dame Australia, Perth, Western Australia, Australia; 3 La Trobe Sports and Exercise Medicine Research Centre, La Trobe University, Melbourne, Victoria, Australia; 4 Australian IOC Research Centre, La Trobe University, Bundoora, Victoria, Australia; 5 School of Medical and Health Sciences, Edith Cowan University, Joondalup, Western Australia, Australia; 6 Department of Public and Occupational Health, EMGO, Amsterdam UMC Locatie VUmc, Amsterdam, Netherlands; 7 Centre for Sport and Social Impact, La Trobe Business School, La Trobe University, Bundoora, Victoria, Australia; 8 Consumer and Community Involvement Program, WA Health Translation Network, The University of Western Australia, Perth, Western Australia, Australia; 9 Department of Physical Therapy, The University of British Columbia, Vancouver, British Columbia, Canada; 10 Centre for Aging SMART, The University of British Columbia, Vancouver, British Columbia, Canada; 11 Sport Injury Prevention Research Centre, Faculty of Kinesiology, University of Calgary, Calgary, Alberta, Canada; 12 Department of Health, Medicine and Caring Sciences, Unit of Physiotherapy, Sport Without Injury Programme (SWIPE), Linköping University, Linkoping, Sweden; 13 Department of Physiotherapy, Podiatry, Prosthetics and Orthotics, La Trobe University, Melbourne, Victoria, Australia; 14 Strength and Power Research Group, School of Medical and Health Sciences, Edith Cowan University, Joondalup, Western Australia, Australia; 15 Exercise Medicine Research Institute, School of Medical and Health Sciences, Edith Cowan University, Joondalup, Western Australia, Australia; 16 Western Australia Police Academy, Joondalup, Perth, Australia

**Keywords:** Injuries, Performance, Prevention

## Abstract

A healthy police force is a key component of a well-functioning society, yet 1 in 20 law enforcement recruits drop out of the recruit training programme due to injury. This drop-out rate has substantial economic and workforce ramifications. In the Western Australia Police Force, one in five recruits suffers a musculoskeletal injury during the recruit training programme, causing time-loss from work. We will now identify the critical elements of an injury prevention intervention and investigate the needs, experiences and suggested solutions to address potential implementation challenges. Our objective is to co-create an intervention with content and context experts, specifically for Western Australia Police Force recruits, to reduce injury prevalence, incidence rates and burden. A mixed-method participatory action research approach will guide intervention cocreation. Phase 1 will include concept mapping and phase 2 will include focus groups. This research will develop an intervention that the Western Australia Police Force can deliver to reduce injury prevalence, incidence rates and burden among recruits. The effectiveness of the intervention in reducing injury burden, economic burden and implementation will be evaluated.

**WHAT IS ALREADY KNOWN ON THIS TOPIC**

- Drop-out due to injury in law enforcement recruit training has substantial economic and workforce ramifications.

**WHAT THIS STUDY ADDS**

- This study will co-create an intervention with content and context experts, specifically to reduce the injury burden in law enforcement recruits.

**HOW THIS STUDY MIGHT AFFECT RESEARCH, PRACTICE OR POLICY**

- This research will develop an intervention that could reduce the injury and economic burden of law enforcement recruit injury.

## Introduction

 A healthy police force is a key component of a well-functioning society, yet 1 in 20 law enforcement recruits drop out of the recruit training programme due to injury.^1^ This drop-out rate has substantial economic and workforce ramifications. In the Western Australia (WA) Police Force, one in five recruits suffers a musculoskeletal injury during the recruit training programme, causing time-loss from work.^2 3^ In a single financial year (2020–2021), the WA Police Force incurred an estimated financial burden of >US$500 000 based on a time-loss burden of >3000 lost workdays (cost calculated based on recruit salary rates for that year in WA).^2^

Throughout their training, WA Police Force recruits have an estimated injury prevalence of 20.1% and an injury incidence rate of 2.00 (95% CIs 1.78 to 2.24) injuries per 1000 training days.^2^ The prevalence and incidence rates are comparable to those observed in other police and military recruit populations worldwide.^3 4^ In addition, police force recruits predominantly experience injuries affecting the lower limb (eg, ankle, knee and hip) similar to the injury locations in military recruits.^2^ However, in contrast to military populations, the majority of the time-loss burden from police force recruits is from joint, ligament, muscle and tendon injury types, as opposed to bone stress injury.^2^

A range of factors predispose Police Force recruits to injury.^5^ However, few were strongly associated with injury and were identified to be supported with very low certainty evidence.^5^ Older age (>30 years) and female sex were the most consistently reported demographic variables associated with an increased injury incidence rate.^5^ Lower baseline cardiorespiratory fitness, measured using running-related muscle performance outcomes (eg, beep test, 1.5 mile run test) was associated with higher injury incidence rates,^5^ as was lower baseline physical endurance capacity (eg, pull-up repetitions to failure, push-up repetitions to failure and sit-up repetitions to failure). No baseline psychological variables have been evaluated as potential risk factors.^5^

In 2022, the WA Police Force conducted its largest recruitment drive to date, funded by the state government, aiming for 300–400 recruits per year.^6^ A new State policy has committed to 1000 recruits entering training per annum.^7^ A key priority of the initiative is to increase the number of women recruits, Aboriginal and Torres Strait Islander recruits and recruits aged over 30 years to improve diversity. While enhanced diversity is important and desired, our research demonstrated that women are 40% more likely to need training modification during their recruit programme due to injury and recruits aged over 30 years were 50% more likely to require such training modification.^2^ The recruitment drive increase is occurring in combination with a lowering of the baseline physical fitness standards for entry. While this may bolster recruitment, for every level lower on a baseline fitness test, there is a 20% increase in the risk of lower-limb and lumbosacral injury.^8^ Unfortunately, these findings suggest that injuries for recruits are likely to increase. The result is a potential for compounding higher injury incidence rates expected with the inclusion of more women and older recruits, highlighting the need for targeted interventions.

### Objective

Our objective is to co-create an intervention with content and context experts, specifically for WA Police Force recruits, to reduce injury prevalence, incidence rates and burden.

### Consumer and industry engagement

This project was co-created in collaboration with consumers (ie, police recruits and people with a lived experience of severe injury from tactical recruit training) and industry (ie, police force physical training and operational personnel). Co-creation is an active process of collaboration between people (industry, consumers, researchers) with shared goals but different expertise and skills throughout the entire research process.^9 10^ The primary driver for this research from the industry group is exemplified in this quote:

“The challenges and complexities associated with recruitment, retention, and maintenance of our future forces amid a decreasing recruitment pool in the civilian population means it is vital that recruits who commence Academy training are given every support for realising its completion and remaining operationally deployable.” (Mr Garth Allen, Physical Performance Manager at the WA Police Force)

The key findings reported by our consumers and industry partners that guided project development were:

- Any mandatory exercise-based injury prevention intervention must be integrated into existing physical training time and not encroach on recruit free time.
- Optional additional exercises would interest some recruits and be valuable if provided using smartphone-accessible platforms, such as an app or webpage.
- Injury prevention interventions should include more than just exercises (eg, nutrition and education).

Our consumer and industry engagement plan for the project will include the ongoing involvement of two advisory groups. The Consumer Advisory Group will include police recruits and people with lived experience of injury from tactical recruit training. The Industry Advisory Group will include Police Force physical training and operational personnel.

## Methods

### Study design

Co-creation will be conducted using a mixed-method Participatory Action Research approach.^11^ This approach is adapted from our previously successful approaches in other injury prevention projects.^12^,^15^ We will use a participatory action approach, guided by our established seven-step intervention development process,^13^ to cocreate a musculoskeletal injury prevention intervention specific to WA Police Force recruits.

### Procedures

An interdisciplinary research committee, including content (health and injury prevention) and context (consumer and industry) experts, has been established with characteristics described in table 1. This group will follow the established intervention development process we have previously described.^13^ Thus, the cumulative expertise from the group includes muscle injury, tendon injury, joint injury (focus on hip and knee), exercise prescription and performance; implementation science, nutrition and lifestyle, health behaviour change, health promotion, police-specific training and police-specific injury consequences.

**Table 1:** Interdisciplinary project research committee

Member	Expertise	Area of focus
Alex Donaldson	Health promotion and injury prevention researcher	Health behaviour change and health promotion
Andrea Bruder	Clinical physiotherapist and researcher	Implementation and qualitative methods
Andrea Mosler	Clinical physiotherapist and researcher	Lower-limb musculoskeletal injury prevention
Brady Green	Clinical physiotherapist and researcher	Lower limb muscle injuries
Carolyn Emery	Injury epidemiology and injury prevention researcher and physiotherapist	Sport-related injury prevention across the lifespan
Clare Ardern	Physiotherapist, researcher, journal editor	Return to sport, codesign and knowledge mobilisation
Debra Langridge	Consumer and community involvement	Consumer and community involvement and cocreation
Evert Verhagen	Epidemiologist and injury prevention researcher	Qualitative research, implementation, injury prevention
G Gregory Haff	Exercise and sport scientist and researcher	Performance testing, training programme design and tactical strength and conditioning
Garth Allen	Industry advisory group chair	Police-specific physical training
Joanne Kemp	Clinical physiotherapist and researcher	Lower limb injury, joint injury and management
Jonathan Hodgson	Nutrition, lifestyle and health researcher	Conduct of nutrition and lifestyle intervention studies
Kay Crossley	Clinical physiotherapist and researcher	injury prevention, implementation, musculoskeletal health, women in sport
Martin Hägglund	Clinical physiotherapist and researcher	Lower-limb musculoskeletal injury prevention and exercise implementation
Mary Kennedy	Implementation science researcher	Exercise implementation
Myles CMurphy	Clinical physiotherapist and researcher	Lower-limb muscle and tendon injury
Simone Radavelli-Bagatini	Nutrition, lifestyle and health researcher	Conduct of nutrition and lifestyle intervention studies
Sophia Nimphius	Exercise and sport scientist and researcher	Knee injury and biomechanics
Vanessa Sutton	Consumer advisory group chair	Lived experience of injury from tactical recruit training

**Figure 1:**
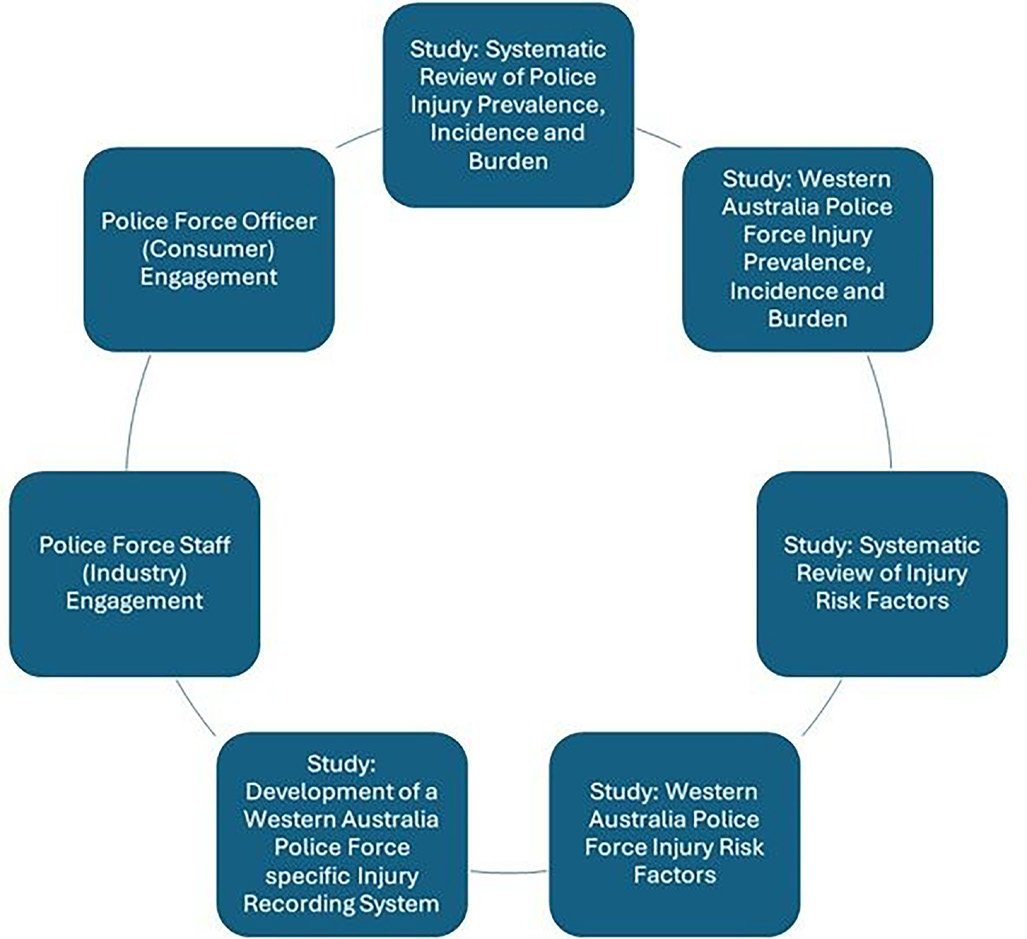
Processes which have informed the current protocol.

We have previously identified and synthesised relevant evidence^2 3 5 8^ and obtained consumer and industry partner input, which has informed this co-creation protocol (figure 1). In this study, we will identify the critical elements of the injury prevention intervention and investigate the needs, experiences and suggested solutions to address potential implementation challenges.

## Phase 1: concept mapping

**Figure 2:**
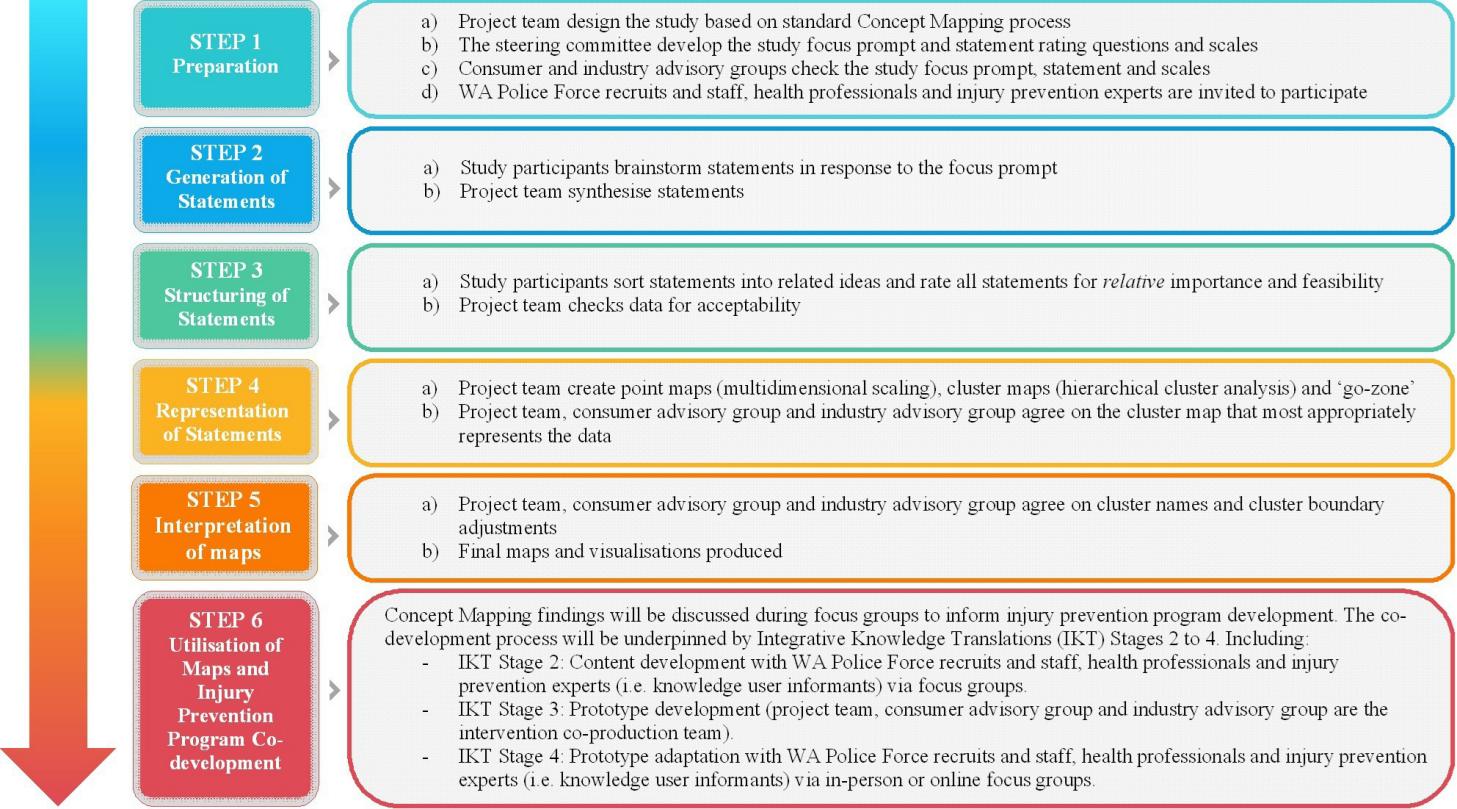
Summary of the co-creation process.

Concept mapping is a six-step mixed-method participatory approach which involves collecting qualitative data that are subsequently analysed quantitatively.^16^ The concept mapping exercise will ask participants to generate and organise ideas in response to study focus prompts (figure 2), including preparation, brainstorming, sorting and rating, analysis, interpretation and utilisation.^17^ Concept mapping is a reliable and valid method to integrate and conceptualise diverse participant perspectives and experiences on a topic of interest.^18^

### Participants

We will recruit approximately 50 participants from distinct groups: WA Police Force recruits; WA Police Force staff; health professionals and injury prevention experts.^13^ The WA Police Force recruits will include adults over 18 years of diverse genders, with lived experience of working in the WA Police Force in the previous 12 months and/or having had an injury during tactical training resulting in an inability or impaired ability to work. The WA Police Force staff will include people who are involved in administering and delivering WA Police Force recruit training (eg, physical trainers, police instructors, health and wellness staff, or executive staff). Health professionals will include those with experience in preventing musculoskeletal injuries (eg, sports medicine physicians, physiotherapists, exercise physiologists, physical trainers and psychologists). Injury prevention experts will include recognised experts with relevant publications in the field of musculoskeletal injury prevention in physically active populations (eg, tactical or sporting populations), with consideration for selection using the closeness continuum.^19 20^ A minimum of 10 participants is typically required to conduct a Concept Mapping study. However, there is no strict limit to the number of people who can participate.^17^ A target of 50 participants is based on our previous research in this field as enabling saturation without receiving an excess of redundant responses.^12 21 22^

### Recruitment

Participants will be recruited in March 2025 via purposive and snowball recruitment strategies, consistent with our desired sample size for concept mapping.^17^ Potential participants will be approached via existing industry networks, professional organisations and social media advertisements. Participants in phase 1 will receive a $A50 gift card as an honorarium.

### Setting

Participants will complete an online Qualtrics survey including several variables to describe the cohort. After completing the survey, participants will receive an email link to the concept mapping exercise. All concept mapping data will be collected and analysed using the Concept Group Wisdom software (Ithaca, New York, USA).

### Demographic data

The following data will be collected using Qualtrics before all participant groups contribute to the concept mapping: Age (years); sex recorded at birth (male/female/another term (please specify)); current gender (man or male/woman or female/non-binary/different term/prefer not to answer); body mass index (calculated from self-reported height and weight); ethnicity (self-reported); country of primary residence (self-reported); languages other than English spoken by the participant (self-reported); whether the participant performs moderate to vigorous physical activity most days (yes/no); highest level of education (less than high school degree/high school graduate/bachelor’s degree/master’s degree/doctoral degree/other), professional degree (eg, medical doctor); employment status (working full-time/working part-time/student/homemaker or stay-at-home parent/unemployed and looking for work/retired/other); total household income (less than $A30 000/between $A30 000 and $A49 999/between $A50 000 and $A79 999/between $A80 000 and $A99 999/between $A100 000 and $A149 999/between $A150 000 and $A199 999/more than $A200 000/prefer not to say); caregiving responsibilities; and Nordic musculoskeletal screening (neck; shoulders; upper back; elbows; wrists/hands; lower back; hips/thighs; knees; ankles/feet).^23 24^ Participants will then be asked to select the most relevant group of interest (WA Police Force staff recruits; WA Police Force staff; health professionals; injury prevention experts), with the inclusion criteria for each group provided. Participants will then self-report their specific occupation (eg, detective, medical doctor and physical trainer) and the number of months and years they have worked in their role. WA Police Force representatives who have left the Police Force will report in months and years how long they were in the WA Police Force. WA Police Force recruits will also report whether they have previously been employed in a tactical athlete role (eg, in the military or other law enforcement agency). Finally, injury prevention experts will also be requested to name the injury prevention programmes and contexts they have experience with (eg, knee injury prevention in soccer, lower back injury prevention in military).

### Concept mapping data

#### Brainstorming

Using the Concept Group Wisdom software, participants will be asked to respond to the study focus prompt: “To prevent injury and/or reduce the impact of injury on law enforcement recruit training, I think it’s important to….” Participants can brainstorm as many statements as they want to and will have access to the software for a 10 day period, with regular reminders. After the brainstorming activity, the steering committee will synthesise the statements via an iterative process. Synthesis will include splitting compound statements, deleting statements unrelated to the study prompt, checking and editing statements to enhance clarity or grammar and removing duplicate or unclear statements.^25^

#### Statement sorting and rating

Following synthesising the brainstorming data, participants will be invited to complete statement sorting and rating over a second 10-day period. Participants will be asked to group the synthesised and randomised statements into 5–15 piles of related ideas and to name each pile based on its theme/contents. Participants will be specifically instructed to avoid grouping statements based on a value (eg, a group of what they think are most important) and to avoid creating groups of unrelated statements (eg, a group containing items they are unsure about). Participants will be asked to rate each statement for importance and feasibility using a 5-point scale (ie, 1=least important, 5=most important). Participants will be specifically instructed to rate each statement relative to other statements on the list and use all item response categories (eg, avoid assigning all items a 1 or 5 only).

#### Analysis and interpretation

Point and cluster maps will be produced using multidimensional scaling and hierarchical cluster analysis (figure 2). The intervention development team will iteratively review the statement groups in the cluster maps in collaboration with our consumer and industry advisory groups. A final cluster solution will be selected when the cluster map best conceptually, statistically and practically represents how the participants sorted the statements. Mean importance and feasibility ratings will be calculated for each statement and used to generate a ‘Go-Zone’, with each statement’s mean ratings plotted on a scatterplot divided into four quadrants using the grand mean of each rating scale to identify priority ideas to address (eg, those that are above the grand mean on both scales or above the grand mean on importance but below the grand mean on feasibility).

## Phase 2: focus groups

The integrated knowledge translation (IKT) approach will guide the focus group structure, concentrating on increasing knowledge use, impact and practicality. This approach is similar to other collaborative research cocreation approaches.^26^ IKT stages 2–4 (figure 2) will include programme content development (stage 2), prototype development (stage 3) and prototype adaptation (stage 4).

### Participants

We will perform two rounds of in-person or online focus groups, which will be facilitated by steering committee members and include representation from WA Police Force recruits, WA Police Force staff and health professional/injury prevention experts (~3 focus groups with 6–8 participants).

### Recruitment

We will invite participants who participated in phase 1 to participate in phase 2, with selections based on ensuring that a sample is as diverse as possible for our focus groups. Participants in phase 2 will also receive a $A50 gift card as an honorarium.

### Setting

In-person focus groups will be conducted at the WA Police Force Academy in Joondalup, WA, for WA Police Force recruits and WA Police Force staff. Health professionals and injury prevention experts who are unable to participate in person will have the opportunity to participate in an online focus group using Microsoft Teams. Individual interviews may be conducted to accommodate participants who cannot attend a focus group.

#### Focus group data and analysis

The objectives of the first round of focus groups (IKT stage 2) include confirming and understanding the intervention components (ie, concept mapping results), identifying implementation barriers and enablers and identifying key resources to enhance intervention delivery.

A short preparatory video will be created by our project team members who have lived experience of injury resulting from tactical performance (VRS) and the WA Police Force physical performance manager (GA). The video will explain the project’s background, aims and impact—encouraging participants to share their ideas and opinions. The video will be played to participants before the focus groups. During the focus groups, the lead facilitator will iteratively summarise and confirm ideas to enhance interpretation accuracy and consensus. All focus groups will be audiovisually recorded, and the co-facilitators will take field notes, with all recordings transcribed electronically. We have attempted to include co-facilitators from various backgrounds with experience in qualitative research to achieve more structured member checking. This way there is diverse input for preliminary themes, ensuring less attention and confirmation bias from facilitators.

A researcher will iteratively code all focus group data using QSR NVivo under the guidance of senior members of the research team who have substantial experience with QSR NVivo for qualitative research analysis.^27 28^ Following Braun and Clarke’s six stages of thematic analysis,^29^,^31^ codes and categories will be generated from data before constructing themes. Initial codes and categories will form focus group summaries that will be distributed to focus group participants for checking.

The project steering committee, research advisory, consumer advisory and industry advisory groups will then integrate concept mapping findings and round one focus group outcomes to develop the injury prevention prototype (IKT stage 3). This will include collaboratively making prototype decisions regarding the content, delivery mode and intervention resources. The prototype will be underpinned by evidence-based theoretical principles (Behaviour Change Wheel ^32^) and mapped to the Template for Intervention Description and Replication checklist to enhance intervention reproducibility.^33^ The final prototype will be provided to focus group participants for checking.

Finally, the participants from our first round of focus groups will participate in a second round of focus groups to adapt the prototype, as needed, and prepare for implementation (IKT stage 4). Feedback will be sought on the prototype itself and the intervention resources drafted. Documents will be sent out to focus group participants with questions to consider before focus groups. The focus group objectives include presenting the injury prevention intervention, discussing modifications to improve intervention acceptability and answering other identified questions.

## Significance

This research will develop an intervention that the WA Police Force can deliver to reduce injury incidence rates and burden among recruits. The effectiveness of the intervention to reduce injury burden, economic burden and intervention implementation will require subsequent evaluation.

## Data Availability

No data are available.
